# Changes in Injury Pattern and Outcomes of Trauma Patients after COVID-19 Pandemic: A Retrospective Cohort Study

**DOI:** 10.3390/healthcare11081074

**Published:** 2023-04-10

**Authors:** Myungjin Jang, Mina Lee, Giljae Lee, Jungnam Lee, Kangkook Choi, Byungchul Yu

**Affiliations:** 1 Department of Trauma Surgery, Gachon University Gil Medical Center, Incheon 21556, Republic of Korea; 2 Department of Traumatology, Gachon University, Incheon 21565, Republic of Korea

**Keywords:** COVID-19, pandemic, trauma center, injury

## Abstract

The COVID-19 pandemic, starting in 2020, changed the daily activities of people in the world and it might also affect patterns of major trauma. This study aimed to compare the epidemiology and outcomes of trauma patients before and after the COVID-19 outbreak. This was a retrospective study, conducted in a single regional trauma center in Korea, and patients were grouped as pre- and post-COVID-19 and compared in terms of demographics, clinical characteristics, and clinical outcomes. A total of 4585 patients were included in the study and the mean age was 57.60 ± 18.55 and 59.06 ± 18.73 years in the pre- and post-COVID-19 groups, respectively. The rate of elderly patients (age ≥ 65) significantly increased in the post-COVID-19 group. In terms of injury patterns, self-harm was significantly increased after COVID-19 (2.6% vs. 3.5%, *p* = 0.021). Mortality, hospital length of stay, 24 h, and transfusion volume were not significantly different. Among the major complications, acute kidney injury, surgical wound infection, pneumonia, and sepsis were significantly different between the groups. This study revealed changes in the age of patients, injury patterns and severity, and major complication rates after the COVID-19 outbreak.

## 1. Introduction

In 2012, the importance of trauma centers in Korea was highlighted by Operation Dawn of the Gulf of Aden, which rescued Captain Seok Hae-gyun [1]. Trauma is the fourth leading cause of death in Korea after cancer and cardio- and cerebrovascular diseases and the most common cause of death in adults under the age of 45, making it a major cause of death in the socioeconomically active age group. Since 2012, the government and medical community have been trying to establish trauma centers. As of 2022, 17 regional trauma centers have been designated and are operating [2,3]. The main objective of this project was to establish regional trauma systems in Korea, including regional trauma centers that are equipped to manage trauma patients based on the standards of level I trauma centers in the United States.

The World Health Organization declared coronavirus disease (COVID-19) a pandemic on 11 March 2020. Governments worldwide have taken action to minimize disease transmission rates and adjust resources to provide care for patients. Policies such as localized lockdowns, social distancing, and self-quarantine were introduced and have influenced the country’s lifestyle, economy, and healthcare. The policies of the Korean government have changed several times. The first wave of COVID-19 in Daegu metropolitan city began in February 2020. Subsequently, strict and detailed social distancing regulations were introduced on 28 June 2020, which included the regulation of facility use and social gatherings [4].

According to the National Emergency Medical Center, in 2021, there were a total of 7,124,677 trauma visits, of which 34,835 were at regional trauma centers. Additionally, 8906 severe trauma patients had an injury severity score of ≥15, representing 25.6% of all trauma patients. In 2019, before the COVID-19 outbreak, 37,635 trauma patients visited regional trauma centers, of whom 8892 (23.6%) had severe trauma. In 2020, after the COVID-19 outbreak, 34,318 patients visited regional trauma centers, of whom 8918 (26.0%) had severe trauma. This showed that there was a slight decrease in the number of patients visiting regional trauma centers, but the percentage of severe trauma patients increased after the COVID-19 outbreak.

Since the first COVID-19 case was detected in Korea at the beginning of 2020, there has been no significant change in the number or severity of trauma patients, even though the overall lifestyle of society has changed, due to social distancing as a national outbreak prevention measure. These results show that the number of medical personnel working in regional trauma centers did not change before or after the COVID-19 outbreak. However, the need to wear protective equipment to prevent infectious diseases, delays in testing, and changes in patient stay times based on COVID-19 test results may have affected the physical and mental stress of dedicated staff and the quality of trauma care [5,6].

Among the studies on trauma patients after the COVID-19 outbreak, most focused on the mechanisms of injury, as well as on changes in the number of surgeries for orthopedic trauma patients, and changes in patient trends for specific diseases and treatment guidelines during the COVID-19 outbreak [7,8,9]. Kim et al. [10] and Chang et al. [11] studied trauma patients before and after the COVID-19 outbreak in Korea. However, most of these studies focused on trauma patients for a short time in the first one to three months after the first case of COVID-19 in Korea, or they defined the difference between pre- and post-COVID-19 as the time when the first case happened before social distancing was actively implemented. In South Korea, strict social distancing measures have been implemented since the second half of 2020. Because of this, it is difficult to explain the differences in the clinical characteristics of trauma patients before and after social distancing measures were implemented.

In this study, we compared the mechanisms of injury, intent, and various clinical characteristics of trauma patients before and after the COVID-19 outbreak. We also analyzed the differences in the rate of complications between trauma patients before (one year in 2019) and after (one year in 2020 to 2021, from 1 June 2020to 31 May 2021) the outbreak to provide up-to-date evidence for the development of treatment guidelines for trauma patients during the COVID-19 outbreak.

## 2. Materials and Methods

### 2.1. Study Design

This retrospective cohort study sought to identify differences in injury types and clinical characteristics of trauma patients before and after the COVID-19 outbreak, as well as in the rate of complications, using data collected from the Korean Trauma Data Bank (KTDB). Gachon University Gil Medical Center is an academic hospital located in Incheon city, South Korea, with a capacity of 1500 beds and serving a population of approximately three million people. The hospital’s trauma center was established as one of the first trauma centers in Korea, with the goal of regionalizing it to a Level I trauma center. The trauma center admits over 3000 patients annually, with more than 500 to 650 patients having an Injury Severity Score (ISS) greater than 15. The center is well-equipped with a trauma bay, two operating rooms specifically dedicated to trauma surgeries, a dedicated 20-bed trauma ICU, and a trauma-interventional radiology suite. The center has more than sixteen full-time trauma surgeons, five trauma coordinators, and eight physician extenders currently working. A team of emergency physicians, anesthesiologists, and residents is stationed at the center, and consultants from neurosurgery and orthopedics are available 24/7. There is no lower-level trauma (similar to level II~IV) center in Korea; instead; there are only regional emergency (highest level) and local emergency (lower level) centers for the management of trauma patients.

The KTDB is a government-developed database, and it is mandatory for every regional trauma center to register data from all trauma admissions, regardless of the ISS. We extracted and analyzed the dataset from KTDB, in which more than 250 variables, including demographics, pre-hospital information, time factors, clinical characteristics, initial, as well as the the worst, vital signs, trauma scores, in-hospital information and outcome information were recorded. The KTDB is prospectively registered by five full-time trauma coordinators, and the average annual number of registration cases is about three-thousand. The reliability and quality of the database are regularly evaluated by agents of the national emergency medical center and errors were corrected by the evaluation report.

### 2.2. Study Population

The subjects of this study were patients who were referred to the regional trauma center and regional emergency medical center of G University Hospital, located in I City, and received a trauma code with a diagnosis code of S or T. The patients were divided into two groups as follows: (1) patients admitted from 1 January 2019 to 31 December 2019, before the start of the COVID-19 outbreak, and (2) patients admitted from 1 June 2020, to 31 May 2021, when the COVID-19 outbreak began and was recognized as a full-fledged global outbreak, with social distancing measures in place. 

A total of 6472 patients were observed during this period, and a total of 4585 patients were included in the final sample, after excluding 1887 who met the exclusion criteria, such as non-trauma patients due to entry errors, patients who were dead on arrival, and patients who were discharged from the emergency department and sent home.

### 2.3. Data Collection and Analysis

Five researchers, including the principal investigator, collected additional data through the EMR after receiving the trauma patient data from the regional trauma center. The variables, including basic demographics, the place of incident, injury mechanism, injury severity score, previous history of medical illness, amount of transfusion and ER stay times, were reviewed. The collected data were analyzed using IBM SPSS Statistics for Windows/WIN, version 25.0 (IBM Corp., Armonk, NY, USA) using the following specific methods: (1) The general and clinical characteristics of the subjects were analyzed using descriptive statistics; (2) clinical outcomes of the group were analyzed using the Chi-squared and independent t-tests; (3) differences in the rate of complications between groups were analyzed using the Chi-squared and multivariate logistic regression tests. 

## 3. Results

### 3.1. Differences in Demographic and Clinical Characteristics between Groups

A total of 4585 patients were included in this study, with 2396 and 2189 in the pre- and post-COVID-19 groups, respectively. The mean age was 57.60 ± 18.55 and 59.06 ± 18.73 years in the pre- and post-COVID-19 groups, respectively, with a significant difference between the groups (t = −2.66; *p* = 0.008). There was also a significant difference in the number of elderly patients aged ≥65, with 36.3% (n = 870) in the pre-COVID-19 group and 40.2% (n = 880) in the post-COVID-19 group (χ^2^ = 7.34; *p* = 0.007).

There were more males than females in both groups, with no significant difference between the groups. Direct admissions comprised 67.2% (n = 1610) and 70.1% (n = 1534) in the pre- and post-COVID-19 groups, respectively, with a significant difference between the groups (χ^2^ = 4.41; *p* = 0.036). In terms of the intention to harm, self-harm had rates of 2.6% (n = 63) and 3.5% (n = 77), whereas violence by others comprised 2.0% (n = 48) and 3.0% (n = 65) of cases, in the pre- and post-COVID-19 groups, respectively, with a significant difference in intention to harm between the groups (χ^2^ = 7.70; *p* = 0.021). Motor vehicle collision (MVC)-related injury was decreased in the post-COVID-19 group without statistical significance (34.5% vs. 30.5%). The rate of pedestrian traffic accident significantly decreased, from 10.4% to 8.6%, in the post-COVID 19 group (χ^2^ = 4.281; *p* = 0.039). In contrast, slips and falls had significantly increased from 29.9% to 33.9% in the post-COVID-19 group (χ^2^ = 8.688; *p* = 0.003). There was no significant difference in the level of consciousness, Glasgow Coma Scale scores, or vital signs in both groups. Differences in Injury severity, measured using an injury severity score (ISS), were significantly in both groups. Minor injuries (ISS < 9) were decreased from 41.3% to 35.4%, but severe injuries (ISS ≥ 16) comprised 22.1% (n = 528) and 24.1% (n = 527) in the pre- and post-COVID-19 groups, respectively, with a significant difference (χ^2^ = 16.86; *p* = 0.001). Vital signs and the revised trauma score were not different in both groups (Table 1).

### 3.2. Differences in Clinical Outcomes between Groups

The 24 h transfusion volume did not differ between the two groups, but massive transfusions were more common in the pre-COVID-19 group than in the post-COVID-19 group (χ^2^ = 6.87; *p* = 0.009). The emergency department length of stay and emergency care outcomes were not significantly different, but the time from the visit to the operating room to angiography was reduced by an average of eight minutes in the post-COVID-19 group. There was no difference in mortality between the two groups, and there was no difference in the total length of stay (Table 2).

The types of complications between the groups were analyzed by focusing on the major complications that affected the major causes of death, lengths of stay in the intensive care unit, and total hospitalizations of major trauma patients (Table 3). Among the major complications, acute kidney injury (χ^2^ = 18.85; *p* <0.001), surgical wound infection (χ^2^ = 4.53; *p* = 0.033), deep vein thrombosis (χ^2^ = 9.53; *p* = 0.002), pneumonia (χ^2^ = 4.52; *p* = 0.034), pulmonary thromboembolism (χ^2^ = 5.35; *p* = 0.021), urinary tract infection (χ^2^ = 10.73; *p* = 0.001), and severe sepsis (χ^2^ = 8.38; *p* = 0.004) significantly differed between the groups.

A multivariate logistic regression analysis evaluated the independent risk factors for complications. as listed in Table 4. After adjusting for age, sex ISS, transfusion, ER stay time, and initial systolic blood pressure, the post-COVID-19 period was significantly associated with the development of complications (odds ratio = 1.81; 95% confidence interval = 1.3–2.51). Being of an age over 65, the male sex and having ISS were also independent risk factors for complication development.

## 4. Discussion

The study found that the age of trauma patients increased by an average of two years after COVID-19, with an increased incidence of injury in patients aged ≥65. Studies by Park et al. [12] and Chang et al. [11], who analyzed the characteristics of trauma patients at the beginning of the COVID-19 outbreak, reported no significant difference in age groups. The mean age in this study was 58 years, which may be due to differences in the regional characteristics of the trauma center and this study, given that the mean age of patients in both studies was in the early 50s. As age is a major factor affecting mortality and the rate of complications in trauma patients, we suggest that future multicenter studies are required to analyze the differences in mortality and the rate of complications adjusted for these age variables more clearly.

The increased rate of direct admissions to the trauma center may be due to the increased percentage of severe trauma patients after the COVID-19 outbreak or because other emergency medical services in the city could not treat trauma patients after the COVID-19 outbreak. In another study, Park et al. [12] found that the number of direct admissions increased, and similar conclusions could be inferred from our findings. So, as the number of direct visits to severe trauma patients increases, regional trauma centers should have the people, materials, and administrative resources to help these patients. The guidelines and instructions for surgery and other major procedures should be prepared to avoid medical gaps [13].

The results showed that the proportion of unintended accidents decreased and the proportion of suicides and intended injuries increased. These results are consistent with the findings of previous studies by Chang et al. [11] and Park et al. [12]. Studies from overseas [14,15,16,17] have also reported a decrease in the proportion of accidents and an increase in the incidence of suicide, injury, and domestic violence. Studies reported isolation and quarantine have aggravated depression and anxiety; at the same time, social distancing rules have caused stress, depression, irritability, insomnia, fear, confusion, anger, frustration, and boredom in confined people with altered daily activities [18]. The increase in suicides and injuries not only endangers the safety and lives of patients but also the safety of medical personnel. Previous studies have shown that nurses working in regional trauma centers report severe mental stress due to the aggression and violence of trauma patients. The stress caused by this anxiety is thought to cause a decrease in work performance and is a major problem in the rapid treatment of severe trauma patients [19]. Therefore, the Korean government and healthcare organizations must prepare measures to deal with suicide and violence due to the increased stress caused by the COVID-19 outbreak, and it is recommended to conduct follow-up studies on the trend of self-harm and injury.

There were no differences in the mechanism of injury, physiological signs, clinical outcomes, length of stay, or mortality of trauma patients before and after the COVID-19 outbreak Regarding the mechanism of injury, the rate of motor vehicle collisions (MVCs) decreased, but there was no statistical significance. Only pedestrian accidents were significantly decreased during the pandemic period. Previous studies in Korea and the United States have also reported a decrease in trauma patients related to MVCs [12,20]. In contrast, the rate of slips increased significantly after pandemic, and we hypothesized that it might be related with increased age in post-COVID-19 group. In summary, our study shows the reduced activities related to industry, and transportation of young people and the relatively increased daily activities of the elderly. The severity of trauma increased, which is consistent with previous studies in Korea and abroad [11,12,21,22]. This suggests that the number and characteristics of trauma patients did not change during the COVID-19 outbreak, but their severity increased, and that medical personnel working in trauma centers may have experienced increased workloads and physical and mental stress due to the COVID-19 outbreak, while treating a similar number of severe trauma patients as that before the COVID-19 outbreak. Therefore, healthcare organizations should provide relevant guidelines for stress management and workload reduction for medical personnel after the end of the COVID-19 outbreak.

In this study, there was a significant difference in the rate of complications in patients with trauma before and after the COVID-19 outbreak. Among these complications, significant differences were found in rates of acute kidney injury, surgical wound infection, deep vein thrombosis, pneumonia, pulmonary embolism, urinary tract infection, and severe sepsis, which are major complications that increase mortality in trauma patients, and this may require revised guidelines on the causes of complications and interventions [23,24]. In logistic regressions, the post-COVID-19 group was an independent risk factor for complication development in this study. Having an age over 65, being of the male sex and having ISS were also independent risk factors for complications. However, the rate of these complications in this study was low compared to the overall trauma population, and further epidemiologic investigation is needed to explain their association with the COVID-19 outbreak. Therefore, multicenter follow-up studies of complications in trauma patients are needed to address these limitations.

In this study, we analyzed data for a one-year period before and after the pandemic for several reasons. Initially, we designed the study as a pilot study, comparing data collected less than a year pre- and post-COVID-19, but we were unable to find significant results due to a small sample size. Therefore, we extended the study period to one year. In the future, we plan to conduct research with more variables that were not included in the database for a prolonged period. Additionally, since our trauma center opened in 2014, we believe that its performance has been relatively stable since 2019. Systematic and strict social distancing policies were introduced in June 2020, and the inoculation programs began in February 2021, allowing us to analyze the effect of relatively pure social distancing during the study period. After the pandemic ends in the near future, we can analyze more complete data, over a prolonged period, and compare trauma patient epidemiology with social distancing levels.

This study has several limitations. First, the retrospective study design might have introduced selection bias. Second, changes in trauma patients after COVID-19 cannot be generalized, because this study is single-institution research. The characteristics of trauma patients can be different by the location of the trauma center. Third, our trauma center is not mature because it is opened in 2014. Trauma patient volume, quality indicators, and outcomes are changing year by year. Some results that did not significantly change in the post-COVID-19 period could be a result of the quality-improvement program in our trauma center. For example, we expected ER stay time to be prolonged after the COVID-19 pandemic, but we could not show a significant difference. Fourth, there were no data related to infectious disease, including COVID-19 infection, in our database. In addition, previous history of mental illness was not collected in the database, so we could not analyze the relationship of suicide or violence to mental illness. Finally, although we adjusted for various known confounders in our analysis, we acknowledge that residual confounding bias have influenced our results. While we attempted to minimize this bias, it is a common limitation of observational studies, and it is difficult to completely eliminate. More advanced statistical techniques, such as propensity score-matching, may be considered in future study. 

## 5. Conclusions

In conclusion, it is clear that the COVID-19 pandemic has had an impact on the patterns of major trauma. The study found that the rate of elderly patients increased significantly after the outbreak, along with an increase in self-harm injuries. Additionally, the rate of injuries caused by motor vehicle accidents decreased in the post-COVID-19 group. While mortality, hospital length of stay, and transfusion volume were not significantly different, there were changes in the rates of major complications, such as acute kidney injury, surgical wound infection, pneumonia, and sepsis.

Overall, these findings suggest that the COVID-19 pandemic has had a significant impact on the epidemiology and outcomes of major trauma. It is important for healthcare providers to be aware of these changes and adapt their treatment strategies accordingly. Further research is needed to fully understand the long-term effects of the pandemic on trauma patients and to develop effective interventions to mitigate these impacts.

## Figures and Tables

**Table 1:** Difference between Groups according to General and Clinical Characteristics.

Variable	Categories	Total (n = 4585)	Pre COVID-19 (n = 2396)	Post COVID-19 (n = 2189)	*x*^2^, t (*p*)
		n (%), M ± SD	n (%), M ± SD	n (%), M ± SD	
Age (year)		58.30 ± 18.65	57.60 ± 18.55	59.06 ± 18.73	−2.659 (0.008)
	<65	2835 (61.8)	1526 (63.7)	1309 (59.8)	7.336 (0.007)
	≥65	1750 (38.2)	870 (36.3)	880 (40.2)	
Gender	Male	2807 (61.2)	1451 (60.6)	1356 (61.9)	0.927 (0.336)
	Female	1778 (38.8)	945 (39.4)	833 (38.1)	
Admission route	Direct	3144 (68.6)	1610 (67.2)	1534 (70.1)	4.410 (0.036)
	Transfer in	1441 (31.4)	786 (32.8)	655 (29.9)	
Trauma type	Accident	4332 (94.5)	2285 (95.4)	2047 (93.5)	7.703 (0.021)
	Suicide	140 (3.1)	63 (2.6)	77 (3.5)	
	Violence	113 (2.5)	48 (2.0)	65 (3.0)	
Injury mechanism	In car TA	482 (10.5)	272 (11.4)	210 (9.6)	3.762 (0.052)
	Bicycle	134 (2.9)	65 (2.7)	69 (3.2)	0.778 (0.378)
	Motorbike	358 (7.8)	188 (7.8)	170 (7.8)	0.010 (0.919)
	Other transportation	85 (1.9)	54 (2.3)	31 (1.4)	4.411 (0.036)
	Pedestrian TA	439 (9.6)	250 (10.4)	189 (8.6)	4.281 (0.039)
	Fall down	873 (19.0)	460 (19.2)	413 (18.9)	0.082 (0.775)
	Slip down	1459 (31.8)	716 (29.9)	743 (33.9)	8.688 (0.003)
	Struck	305 (6.7)	160 (6.7)	145 (6.6)	0.005 (0.942)
	Penetrating	218 (4.8)	116 (4.8)	102 (4.7)	0.083 (0.773)
Mental status	Alert	3813 (83.2)	1984 (82.8)	1829 (83.6)	1.733 (0.630)
	Verbal	327 (7.1)	168 (7.0)	159 (7.3)	
	Pain	255 (5.6)	143 (6.0)	112 (5.1)	
	Unresponsive	190 (4.1)	101 (4.2)	89 (4.1)	
ISS	<9	1765 (38.5)	989 (41.3)	776 (35.4)	16.855 (0.001)
	9–15	1765 (38.5)	879 (36.7)	886 (40.5)	
	16–24	602 (13.1)	296 (12.4)	306 (14.0)	
	≥25	453 (9.9)	232 (9.7)	221 (10.1)	
RTS		7.654 ± 0.6280	7.6464 ± 0.6458	7.6642 ± 0.6080	−0.931 (0.352)
GCS		14.28 ± 0.03	14.27 ± 2.60	14.29 ± 2.28	−0.178 (0.858)
Vital signs	sBP (mm/Hg)	142.77 ± 30.96	142.50 ± 30.69	143.07 ± 31.25	−0.615 (0.538)
	≤90	185 (4.1)	97 (4.1)	88 (4.1)	0.003 (0.954)
	>90	4311 (95.9)	2251 (95.9)	2060 (95.9)	
	PR (rate/min)	86.90 ± 18.32	86.89 ± 18.06	86.91 ± 18.60	−0.043 (0.966)

ISS: Injury Severity Score, RTS: Revised Trauma Score, GCS: Glasgow Coma Scale, sBP: Systolic Blood Pressure, PR: Pulse Rate.

**Table 2:** Difference between Groups according to Clinical Outcomes.

Variable	Categories	Total (n = 4585)	Pre COVID-19 (n = 2396)	Post COVID-19 (n = 2189)	*x*^2^, t (*p*)
Transfusion	pRBC < 24 h unit, mean ± SD	5.07 ± 6.66	5.36 ± 6.20	4.75 ± 7.14	1.186 (0.236)
	pRBC > 10 unit/day n (%)	85 (12.7)	56 (16.0)	29 (9.2)	6.869 (0.009)
ER stay time(min)	mean ± SD	250.4 ± 176.1	253.5 ± 171.0	247.0 ± 181.4	1.233 (0.218)
	<90, n (%)	652 (14.2)	265 (40.6)	387 (59.4)	41.088 (<0.001)
	≥91, n (%)	3933 (85.8)	2131 (54.2)	1802 (45.8)	
ER outcome n (%)	Send to ward	2847 (62.1)	1493 (62.3)	1354 (62.1)	0.533 (0.912)
	Send to ICU	1152 (25.1)	605 (25.3)	547 (25.0)	
	Send to OR	523 (11.4)	266 (11.1)	257 (11.7)	
	Death	63 (1.4)	32 (1.3)	31 (1.4)	
Time to OR	Minutes, mean ± SD	136.41 ± 107.31	140.24 ± 113.42	132.44 ± 100.66	0.831 (0.406)
Mortality n (%)	Death	274 (6.0)	142 (5.9)	132 (6.0)	0.022 (0.882)
	Survival	4311 (94.0)	2254 (94.1)	2057 (94.0)	
LOH (days)	mean ± SD	14.59 ± 16.91	14.63 ± 18.10	14.55 ± 15.50	0.172 (0.863)

pRBC: Packed Red Blood Cells, OR: Operation Room, LOH: Length of Hospital Stay.

**Table 3:** Complication rate between the two groups.

Variable	Categories	Total (n = 4585)	Pre COVID-19 (n = 2396)	Post COVID-19 (n = 2189)	*x*^2^ (*p*)
Complications	AKI	66 (1.4)	17 (0.7)	49 (2.2)	18.848 (<0.001)
	ARDS	7 (0.2)	2 (0.1)	5 (0.2)	1.576 (0.209)
	Surgical wound infection	53 (1.2)	20 (0.8)	33 (1.5)	4.532 (0.033)
	DVT	42 (0.9)	12 (0.5)	30 (1.4)	9.532 (0.002)
	Pneumonia	130 (2.8)	56 (2.3)	74 (3.4)	4.520 (0.034)
	PTE	14 (0.3)	3 (0.1)	11 (0.5)	5.350 (0.021)
	UTI	46 (1.0)	13 (0.5)	33 (1.5)	10.725 (0.001)
	CRBSI	12 (0.3)	7 (0.3)	5 (0.2)	0.178 (0.673)
	Sever sepsis	20 (0.4)	4 (0.2)	16 (0.7)	8.378 (0.004)

AKI: Acute Kidney Injury, ARDS: Acute Respiratory Distress Syndrome, DVT: Deep Vein Thrombosis, PTE: Pulmonary Thromboembolism, UTI: Urinary Tract Infection, CRBSI: Catheter-Related Blood Stream Infection.

**Table 4:** Logistic regression analysis of the independent risk factors of complications.

Variables	Odds Ratio	95% Confidence Interval	*p*-Value
Post-COVID-19	1.81	1.3–2.5	<0.001
Age ≥ 65	2.39	1.7–3.37	<0.001
Male	1.75	1.21–2.54	0.003
Lowest SBP	1.47	0.84–2.57	0.177
Transfusion	1.503	0.99–2.28	0.055
ISS	1.08	1.06–1.1	<0.001
ER stay time	0.999	0.998–1	0.136

SBP: Systolic Blood Pressure; ISS: Injury Severity Score; ER: Emergency Room.

## Data Availability

The data presented in this study are available on request from the corresponding author.
